# Novel *EYA4* variant in Slovak family with late onset autosomal dominant hearing loss: a case report

**DOI:** 10.1186/s12881-019-0806-y

**Published:** 2019-05-17

**Authors:** Lukas Varga, Daniel Danis, Martina Skopkova, Ivica Masindova, Zuzana Slobodova, Lucia Demesova, Milan Profant, Daniela Gasperikova

**Affiliations:** 1 Department of Otorhinolaryngology – Head and Neck Surgery, Faculty of Medicine and University Hospital, Comenius University, Bratislava, Slovakia; 2 Diabgene Laboratory, Biomedical Research Center, University Science Park for Biomedicine, Slovak Academy of Sciences, Bratislava, Slovakia

**Keywords:** Sensorineural deafness, Postlingual, Next-generation sequencing, Autosomal dominant

## Abstract

**Background:**

Progressive bilateral sensorineural deafness in postlingual period may be linked to many different etiologies including genetic factors. Identification of the exact deafness cause may, therefore, be quite challenging. Here we present a family with late-onset hearing loss as an autosomal dominant trait caused by a novel *EYA4* mutation.

**Case presentation:**

Forty-four years old female proband clinically investigated for progressive hearing loss and occasional dizziness with positive family history for deafness was subject to molecular-genetic testing. Patient’s DNA sample was analyzed by whole exome sequencing. We identified a novel missense variant c.804G > C located at the last base pair of exon 10 in *EYA4*. Candidate variant was confirmed by Sanger sequencing in the proband and her family members. In silico prediction tools and co-segregation analysis were used to indicate pathogenicity of the identified variant. To confirm our hypothesis, we performed minigene assay to demonstrate if the transcript of exon 10 in *EYA4* is present. We provide evidence that this mutation in vitro compromises donor site functionality and causes exon 10 skipping and frameshift that most likely results in nonsense-mediated mRNA decay. The onset of moderate to severe hearing loss in the family ranged from 10 to 40 years. The normal cardiac phenotype was confirmed by ECG and echocardiography.

**Conclusions:**

We identified a novel *EYA4* mutation associated with adult-onset autosomal dominant sensorineural hearing loss. This report extends the knowledge of spectrum of *EYA4* mutations and demonstrates the pathogenicity of a variant affecting specific position in the gene. A comprehensive review of known *EYA4* mutations is also given and their impact on cardiac phenotype is discussed. Our findings highlight the importance of genetic testing and complex clinical assessment in patients with familial progressive hearing loss.

## Background

Autosomal dominant sensorineural hearing loss (ADSNHL) accounts for about 20% of all hereditary nonsyndromic SNHL cases. Currently, at least 38 genes are known to be associated with nonsyndromic ADSNHL [1]. In contrast to the autosomal recessive SNHL, which is typically congenital or prelingual, the onset of ADSNHL is often delayed and escapes the neonatal hearing screening. It may develop as late as in adulthood and it may even overlap with presbycusis [2, 3]. Therefore, genetic etiology may not raise sufficient awareness in the diagnostic workup. The course of hearing loss in ADSNHL is generally progressive, although the speed of hearing deterioration and its severity may vary among different genes or even among different affected individuals. More importantly, progressive bilateral SNHL is often the first symptom in a number of syndromic forms of hearing loss [4].

One of these genes is *EYA4* (**Ey**es **A**bsent Homolog **4**) which encodes a 640-amino-acid protein that serves as a transcription coactivator. In higher animals *EYA4* is a component of network composed of genes belonging to the Pax, Six, Eya, and Dach families which play a key regulatory role in the development of multiple organs including the eye, muscle, ears, heart, lungs, endocrine glands, placodes, pharyngeal pouches, craniofacial skeleton, and parathyroid [5]. This protein contains a highly conserved 271 amino acid Eya domain (eya homologous region, eya-HR) at the C-terminus and a poorly conserved proline-serine-threonine (PST)-rich transactivation domain called variable region (eya-VR) at the N-terminus [6]. It has been proposed that truncations of the C-terminal Eya domain cause nonsyndromic ADSNHL (DFNA10) whereas upstream truncations deleting the N-terminal variable region cause hearing loss with dilation cardiomyopathy [7]. To date, there are only a few reported families in who ADSNHL segregated with *EYA4* mutations.

Here we present a Slovak pedigree with late-onset progressive SNHL analyzed by whole exome sequencing (WES), which allowed us to identify a novel pathogenic variant in the *EYA4* gene.

## Case presentation

### Diagnostic assessment

#### Family and clinical evaluation

Forty-four-year-old Caucasian female proband suffering from progressive bilateral SNHL since the second decade of her life and positive family history for hearing loss was referred to the Department of ORL-HNS at the University Hospital in Bratislava. Detailed family history questioning revealed other five affected family members in three generations with autosomal dominant inheritance pattern. After excluding the DFNB1 etiology in the proband as a routine step in our diagnostic pipeline for hereditary hearing loss, peripheral blood was taken for DNA analysis and general ENT examination together with audiological tests (tympanometry, stapedial reflexes, ABR, pure tone audiometry in frequency range 250–6000 Hz) were performed in all affected and unaffected family members, who agreed to participate. Additionally, the affected individuals were also subject to vestibular examination (VEMPs, video Head Impulse Test, videonystagmography, caloric testing, and postural tests) to evaluate the vestibular function of the inner ear. Moreover, the proband underwent imaging studies (temporal bone CT and MRI).

After WES results were obtained and hearing loss etiology was determined, three affected subjects older than 40 years underwent detailed cardiological assessment including ECG and echocardiography. All participants or their legal representatives signed informed consent and the study was approved by the Ethics Committee of University Hospital in Bratislava.

#### Whole exome sequencing

Genomic DNA was isolated from peripheral blood using standard procedures. WES was done by a service provider (BGI, HongKong). DNA library was prepared using BGI 59 M Human Exome kit and was sequenced on Complete Genomics Black Bird platform (BGI, Shenzen, China).

Sequencing data was processed by BGI’s standard bioinformatics pipeline which encompassed base calling, alignment of generated reads to the GRCh37 reference genome without the unplaced or alternate loci and variant calling. Aligned reads and called variants were obtained in standard bioinformatics formats and subjected to following bioinformatics pipeline. Variants were decomposed and normalized using vt [8]. Variant effect predictor [9] was used to annotate the variants with respect to their potential effects on genes and transcripts and to add scores from *in-silico* prediction algorithms PolyPhen and SIFT. As the last step of variant annotation, the Gemini framework [10] was used to insert variants into newly created SQLite database and to add additional data from genome annotation databases like dbSNP [11], ENCODE [12], ClinVar, 1000 Genomes [13], Exome Sequencing Project (ESP) [14] and Exome Aggregation Consortium (ExAC) [15].

Then, variant prioritization and filtering were realized by removing those with a MAF ≥ 0.01 (based on dbSNP v138, ESP, ExAC v0.3) and the resulting variant set was further narrowed down by removing variants not lying inside regions of 91 genes with known association with nonsyndromic sensorineural hearing loss (NSNHL) in human. Set of evaluated NSNHL genes was based on the Hereditary Hearing Loss webpage [1].

#### In silico analysis

Functional consequences of candidate variants were evaluated using CADD, PolyPhen and SIFT prediction algorithms [16–18]. The strength of *wt* and mutated splice donor site was assessed using MaxEntScan, Human Splicing Finder and NNSplice [19–21].

#### Sanger sequencing

Sanger sequencing was performed to verify variants identified by WES and to determine co-segregation of the candidate variant with hearing loss in all participating family members. PCR primers amplifying exon 10 and 100 bp of surrounding intronic regions of *EYA4* were designed using Primer BLAST software. Sequencing reactions of PCR products were carried out using BigDye Terminator v3.1 chemistry and separated on ABI 3500 genetic analyzer (Applied Biosystems) according to manufacturer’s instructions. GenBank RefSeq NM_004100.4 was used as the *EYA4* reference sequence.

#### Minigene assay

Minigene assay was performed as described in [22]. The pSpliceExpress vector was a gift from Stefan Stamm (Addgene plasmid # 32485). Briefly, *EYA4* exon10 was amplified from patient DNA using primers with attB1 and attB2 tails and was cloned into the pSpliceExpress using Clonase BP (Invitrogen). Competent cells (SIG10 5α, Sigma) were transformed and positive clones were selected on ampicillin (100 mg/ml). The sequence of selected clones was verified using colony PCR and sequencing. Purified plasmids from two clones, one wild type and one carrying tested mutation, were used for lipofection of HeLa (Sigma) cells. Cells were lysed after 24 h, RNA was extracted with DNaseI step included, reverse transcribed, the minigene cDNA was amplified, and presence/absence of *EYA4* exon10 was tested on agarose gel electrophoresis and verified by sequencing. Quantification of band densities was carried out with ImageJ [23] and counted ratio was corrected for the molecular weight of compared fragments.

### Clinical findings

**Fig. 1:**
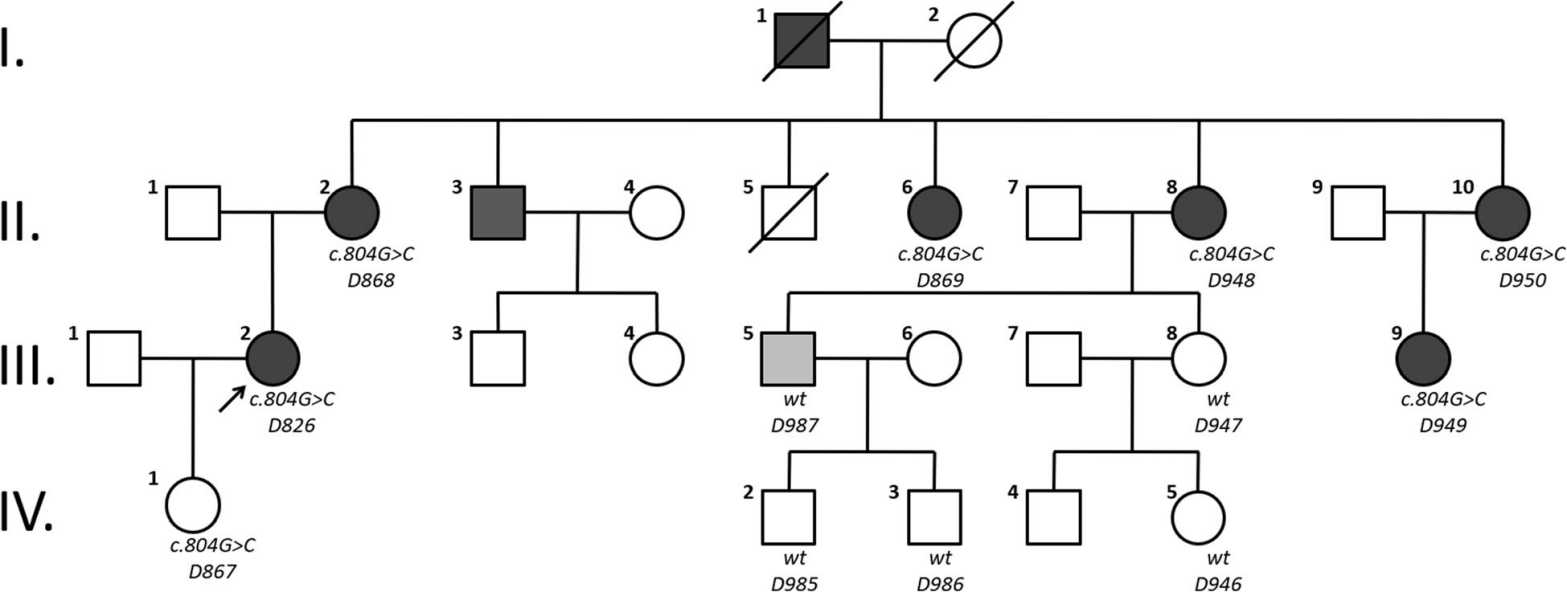
Pedigree of the family depicting segregation of c.804G > C. Arrow indicates the proband where WES was performed. Squares indicate males, circles females. White symbols stand for unaffected family members, filled with black denote affected individuals, grey indicates unilateral hearing loss

**Fig. 2:**
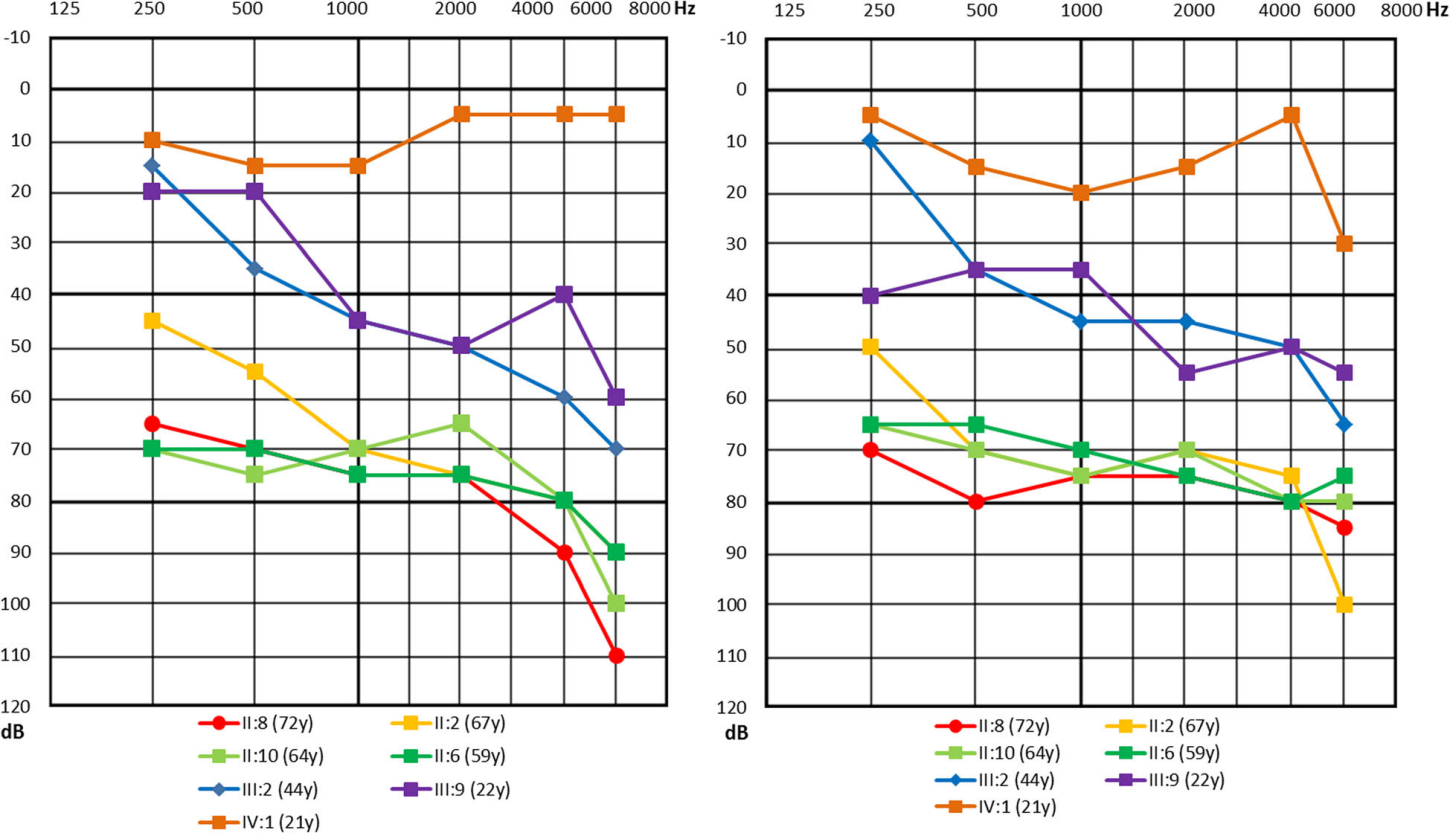
Pure tone audiograms of the affected family members. Left chart – right ear, right chart – left ear. The number in the parentheses corresponds to the subject’s age at audiometric testing in this study

**Fig. 3:**
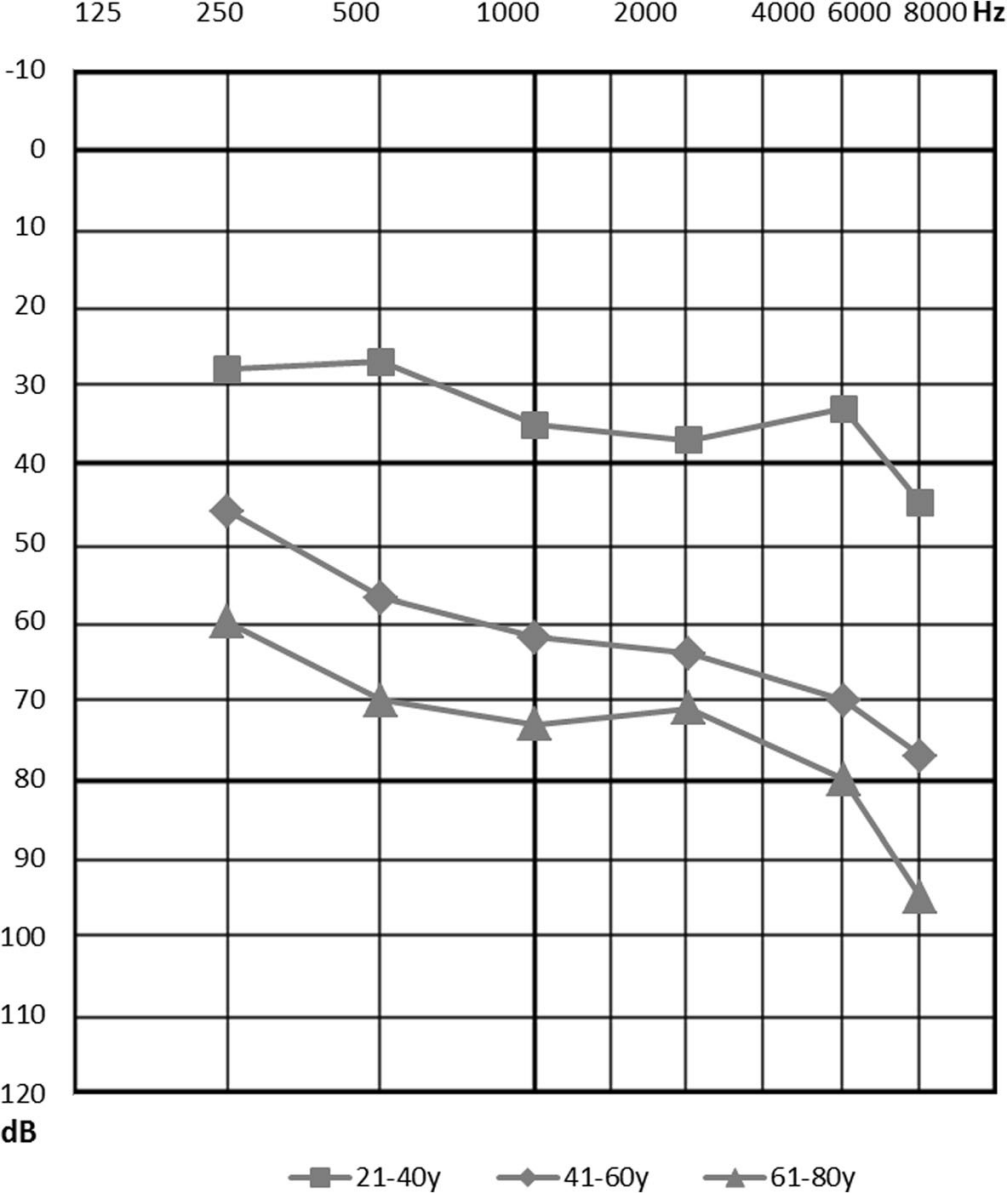
Age-related typical audiogram demonstrating the progression of hearing loss in different life decades

The pedigree of the family presenting with autosomal dominant NSNHL is shown in Fig. 1. In total, 8 affected individuals in 4 generations could be identified presenting with postlingual bilateral progressive sensorineural hearing loss. From 12 individuals available for genetic and audiological testing we identified 6 subjects with bilateral sensorineural hearing loss corresponding to the phenotype of the proband. In female subject III:9 this was the primary diagnosis of hearing loss, as she was not aware of any major subjective hearing problems yet. The degree of hearing loss in the affected subjects ranged from moderate to severe hearing loss based on pure tone average at frequencies 500, 1000, 2000 and 4000 Hz (Fig. 2). Moreover, one subject (III:5) demonstrated unilateral SNHL. The age of hearing loss onset varied between 10 and 40 years and had a progressive course in all subjects based on serial audiograms. One mutation carrier (IV:1) had yet normal pure tone thresholds at the age of 21 years. Construction of age-related typical audiogram (ARTA) showed the most rapid hearing deterioration in the 5th and 6th decade of life (Fig. 3). All subjects requiring hearing rehabilitation benefited from conventional hearing aids. Tinnitus was inconstantly present in 3 subjects, without any association to age or hearing loss severity. Vestibular symptoms (vertigo with occasional headache) only occurred in the proband. Based on the clinical history and results of vestibular testing we supposed the diagnosis of vestibular migraine independently from the etiology of hearing loss. Temporal bone anatomy in the proband was normal based on CT and MRI scans.

Cardiologic findings demonstrated normal heart morphology and function in all three investigated subjects (II:2, II:6, III:2), without any signs of dilation cardiomyopathy. Similarly, no history of sudden cardiac death or increased prevalence of cardiac disease was recorded in the family. Additional clinical findings, which were only present in the mutation carriers, but did not clearly cosegregate with the mutation included blindness in subject II:10 due to bilateral retinopathy with onset in childhood.

### DNA analysis results

Whole exome sequencing was performed in the proband (III:2). In total, 558 million uniquely mapped reads with MAPQ ≥30 were generated, covering 93.45% of exome target regions at least 20x. The overall mean sequencing depth was 162.22x (Table 1). A total of 46,457 variants were called, transition vs. transversion ratio and values of heterozygous vs. non-reference homozygous genotype ratios were within recommended boundaries for WES data [24, 25]. Of those, 482 variants were novel and 1348 variants were present in at least one SNP polymorphism database (dbSNP, ESP, ExAC) with minor allele frequency (MAF) less than 1%. Finally, 19 rare/novel variants were found inside regions of 91 genes which had been associated with NSNHL before.

**Table 1:** Summary of whole exome sequencing and variant analysis

Summary of whole exome sequencing data	
Subject	III:2
Sequencing, read alignment and coverage	
Uniquely mapped reads with MAPQ > = 30 [N]	558,548,129
Fraction of targets covered > = 5x [%]	98.35
Fraction of targets covered > = 20x [%]	93.45
Overall mean sequencing depth [x]	162.22
Variant calling	
Total identified variants [N]	46,457
Known variants with MAF < 0.01 (dbSNP,ESP,ExAC) [N]	1348
Novel variants [N]	482
Heterozygous/non-reference homozygous ratio	1.64
Transition/transversion ratio	2.65

Ultimately, only 5 from 19 variants left by upstream analysis were localized within coding regions of NSNHL genes and only 3 variants were predicted to result in a change of amino acid sequence (Table 2). The only novel variant was *EYA4*: c.804G > C transversion, which in respect to the amino acids is inferred to replace the glutamine with histidine residue at position 268 in the eyes absent 4 protein. However, since the changed nucleotide is the last base of *EYA4* exon 10, and similar variants surrounding splice sites in other genes can induce changes in mRNA splicing [26, 27], additional analysis using *in-silico* splice site prediction tools was performed.

**Table 2:** Rare and novel variants present in coding regions of 91 genes associated with non-syndromic hereditary hearing loss

Rare and novel variants present in panel of NSNHL genes in proband										
Gene	Nucleotide	Exon	rsID	Sequence impact	CADD scaled	PolyPhen	SIFT	MAF 1000G all	MAF ESP all	MAF ExAC all
*USH2A* NM_206933.2	c.14074G > A p.Gly4692Arg	64	rs45549044	Missense	19.61	PD	T	0.002	0.0048	0.0048
*EYA4* NM_004100.4	c.804G > C p.Gln268His	10	N/A	Missense, Splicing region variant	25	PD	D	N/A	N/A	N/A
*MCPH1* NM_024596.4	c.2180C > T p.Pro727Leu	12	rs199861426	Missense	13.36	B	D	N/A	0.0008	0.0011
*MYO7A* NM_000260.3	c.5598C > A p.Leu1866=	40	rs111033504	Synonymous	3.5	N/A	N/A	0.002	N/A	0.0022
*OTOA* NM_144672.3	c.2229C > T p.Ala743=	20	rs461179	Synonymous	1.96	N/A	N/A	N/A	N/A	0.0094

*1000G* 1000 Genomes project, *ESP* Exome sequencing project, *ExAC* Exome Aggregation Consortium, *MAF* Minor allele frequency, *PD* probably damaging, *B* benign, *T* tolerated, *D* deleterious

The Human Splicing Finder algorithm score was near the threshold value for exon being recognized by the spliceosome, whereas MaxEntScan with NNSplice indicated that the mutated sequence is not likely to be functional splice donor site (Table 3). In combination, these scores suggested a high likelihood of splicing modification with resulting deleterious impact on protein sequence.

**Table 3:** Results from *in-silico* analysis of the splice donor site of the intron 10. Scores for wild-type and c.804G > C sequences are presented

Splice site prediction for splice donor variant c.804G > C			
Program	*wt* score	c.804G > C score	threshold value
MaxEntScan	8.73	−4.91	3
HSF 3.0	77.1	66.08	65
NNSplice 0.9	0.87	splice site not recognized	0.4

A sequence is predicted to be a functional splice site if the score is higher than the given threshold value

**Fig. 4:**
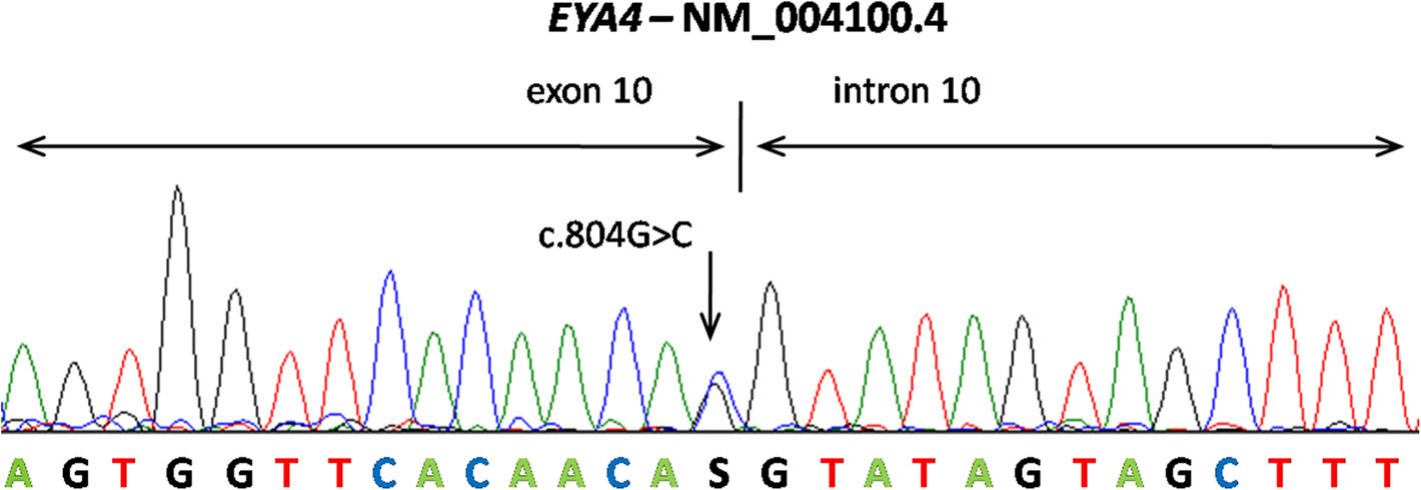
Validation of candidate mutation by Sanger sequencing. The sequence of splice donor site of exon 10 presenting heterozygous mutation c.804G > C in an affected family member (III.2). The arrow indicates the site of mutation

Presence of the c.804G > C variant in proband’s DNA and its heterozygous genotype were confirmed by Sanger sequencing (Fig. 4). Additionally, DNA from all available affected and unaffected family members (*n* = 12) was investigated by cosegregation of the variant with hearing loss. Together 6 relatives of the proband were shown to have the *EYA4* c.804G > C variant, all of them diagnosed with bilateral hearing loss, except individual IV:1, the youngest mutation carrier (21 years), who had yet normal hearing based on pure tone thresholds (Fig. 2). This variant was not present in the DNA of unaffected family members older than 30 years.

#### Functional evaluation of the variant effect on splicing

**Fig. 5:**
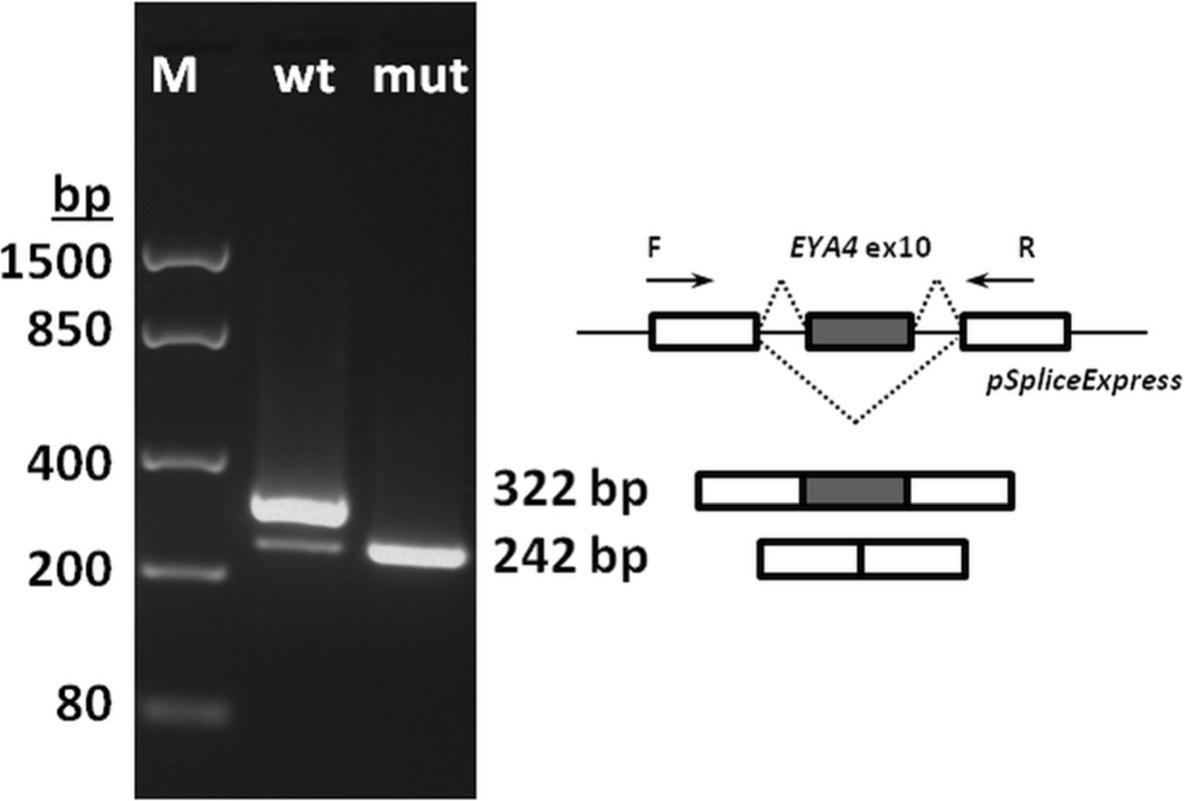
Mini-gene assay results. Wild-type *EYA4* exon 10 (wt) is incorporated into the mature transcript, exon 10 with c.804G > C mutation (mut) is spliced out

The effect of the c.804G > C variant on splicing was tested using minigene assay. The results showed that the wild type exon was correctly incorporated in 89% of transcripts, while the exon carrying the c.804G > C variant failed to be retained in the mature transcript and was cut out during splicing (Fig. 5).

## Discussion & Conclusions

In the present study, we report a four-generation Slovak family in which postlingual nonsyndromic autosomal dominant SNHL segregates with the c.804G > C mutation in *EYA4* discovered using whole exome sequencing approach. The mutation is located at the last base position of the exon 10. To the best of our knowledge, this is the first point mutation localized in exon 10 and the first known case of *EYA4* gene associated with SNHL in the Slavic Caucasian population.

Nucleotide substitutions affecting the last base pair of exon should be regarded as a rare event of which the effect is difficult to predict. At least 10 similar nucleotide changes have been shown to cause abnormal mRNA splicing in mammalian genes [28]. *In-silico* analysis using state-of-the-art prediction algorithms suggests that the mutation weakens splice donor site of intron 10. This may lead to complete or partial skipping of the exon 10, or to the activation of a cryptic splice donor site. As it is known that *EYA4* is not expressed in blood [29], we tried to amplify *EYA4* by RT-PCR from a urine sample from proband as well as from healthy control to determine the exact impact of the mutation of the last exonic nucleotide on mRNA splicing. However, we did not detect any *EYA4* transcripts in the urine sample. Therefore, we applied the mini-gene assay to test the functionality of the exon 10 mutated splice site. Our findings confirmed the *in-silico* predictions and showed that the c.804G > C mutation compromises donor site functionality and causes exon 10 skipping in vitro. Exon 10 skipping in the *EYA4* mRNA splicing would result in frame-shift p.(Ser243Leufs*29) that introduces premature termination codon (PTC) and would thus lead to nonsense-mediated mRNA decay (NMD). However, we cannot exclude the formation of other mutant transcripts, as disruption of one splice site can influence also splicing of other adjacent exons and this we have not tested.

To date, 19 pathogenic or probably pathogenic *EYA4* variants associated with non-syndromic autosomal dominant SNHL (DFNA10) have been reported in about 20 families. Moreover, a large deletion c.581_804del was associated with SNHL and dilation cardiomyopathy resulting in risk of premature death [30, 31]. Genetic and clinical characteristics of known *EYA4* mutations are summarized in Table 4. Similar syndromic phenotype (hearing loss and cardiac malformation) plus microcephaly and mental retardation were observed in 9 Mb deletion 6q23.2–24.1 which also disrupts *EYA4* gene [32].

**Table 4:** List of pathogenic variants in EYA4 identified to date and their hearing loss phenotypes

Exon	Recommended mutation nomenclature*	Amino acid	Variant type	Origin	Age at HL onset	HL degree	Audiogram shape	Variant reported as	Reference
8	c.464delC	p.(Pro155Glnfs*43)	frameshift	Dutch	childhood	moderate	mid- or high-frequency	c.464del	[41]
8	c.511 G > C	p.(Gly171Arg)	missense	Chinese	26–33 years	moderate to severe	gently sloping	c.511 G > C	[42]
8	c.544_545insA	p.(Ser182Tyrfs*63)	nonsense	Chinese	20–40	moderate to profound	high-frequency to flat	c.544_545insA	[43]
8	c.579_580insTACC	p.(Asp194Tyrfs*52)	frameshift	Swedish	4–40 years	mild to profound	variable	c.579_580insTACC	[44]
9–10	c.581_804del	p.(Asp194Glyfs*30)	frameshift	N/A	from schoolage	moderate to profound	mid-frequency	4846-bp deletion	[31]
10	c.804G > C	p.(Gln268His)	missense	Slovak	10–40 years	mild to severe	gently sloping	c.804G > C	this paper
11	c.863 C > A	p.(Ser288*)	nonsense	Korean	N/A	moderate	reverse U-shaped	c.863 C > A	[45]
11	c.978C > G	p.(Phe326Leu)	missense	Korean	N/A	moderate	down-sloping	c.909 C > G	[46]
12	c1026_1027dupAA	p.(Thr343Lysfs*62)	frameshift	North American Caucasian	1st-3rd decade	moderate to profound	gently sloping to flat	c.1468insAA	[36]
12	c.1048_1049dupAA	p.(Arg352Profs*53)	frameshift	North American Caucasian	2nd -4th decade	moderate to severe	mid- to high-frequency	c.1490insAA	[7]
13	c1115_1118dupTTTG	p.(Trp374Cysfs*6)	frameshift	Hungarian	postlingual	up to profound	variable	c.1558insTTTG	[40]
13	c.1154C > T	p.(Ser385Leu)	missense	Italian	postlingual	mild to profound	mid-frequency	c.1154C > T	[47]
13	c.1177C > T	p.(Gln393*)	nonsense	Korean	N/A	moderately severe	mid- and high-frequency	c.1177C > T	[48]
14	c.1194delT	p.(Met401Trpfs*3)	frameshift	Korean	from 1st decade	moderate	down-sloping	c.1194delT	[34]
intron 14	c.1282-12 T > A	splicing effect	splice site	Australian Caucasian	6 years to 4th decade	mild to profound	mid-frequency to flat	c.1282-12 T > A	[38]
15	c. 1301 T > A	p.(Ile434Lys)	missense	Chinese	8–38 years	mild to severe	mid-frequency to flat	c. 1301 T > A	[49]
18	c.1643C > G	p.(Thr548Arg)	missense	Chinese	17–40 years	mild to profound	variable	c.1643C > G	[50]
20	c.1759C > T	p.(Agr587*)	nonsense	Belgian	6–40 years	mild to moderate	mid-frequency	c.2200C > T	[36]

*Nucleotide numbering refers to the EYA4 cDNA sequence NM_004100.4

The most frequent type of mutations observed so far in *EYA4* were frameshifts and nonsense mutations, both leading to the introduction of a premature termination codon (PTC) into mRNA sequence. Presence of the PTC in the mRNA molecule makes the molecule a potential subject of NMD pathway that selectively degrades transcripts carrying PTCs which are 50–55 nucleotides upstream from the last exon-exon junction [33]. In result, mRNA molecule transcribed from the mutated allele would be completely degraded. In the remaining families, nonsynonymous mutations leading to substitutions of highly conserved amino acid residues were identified (Table 4). The prevalence of *EYA4*/DFNA10 related deafness is not known and it was only detected in Caucasian and East Asian population to date. The only relevant data on epidemiology is from Korea, where the prevalence of *EYA4* among nonsyndromic ADSNHL is estimated at 7.4% [34]. In our series, *EYA4* mutation was identified in 5.56% (1/18) of probands with nonsyndromic ADSNHL analyzed by next-generation sequencing.

The exact molecular pathomechanism of hearing impairment associated with mutations in *EYA4* has not been determined yet, but it may result from lowered gene dosage or reduced protein activity. The EYA proteins are transcriptional coactivators that interact with the transcription factors SIX and DACH but lack a DNA-binding domain [35]. *EYA4* acts as a histone phosphatase and promotes efficient DNA repair. Studies of expression in rodent inner ear suggest developmental role during maturation of the inner ear as well as survival role in the mature system [36]. One of the possible pathomechanisms involved in hearing loss development due to *EYA4* mutations may be the impaired regulation of Na+/K + -ATPase by altered expression of its β2b subunit as demonstrated in zebrafish model [37].

Hearing loss in the investigated family had postlingual onset from 10 to 40 years, which corresponds to the onset range (6–40 years) reported in the literature [38]. This relatively broad interval was observed even among the same mutation carriers and members of the same families and is not yet explained. Environmental or intrinsic factors, such as chronic noise exposure or other genetic factors may play a role. However, self-reported hearing loss onset used in this and previous studies is also a matter of subjective disability evaluation, which may vary among affected subjects. Especially in mild degree cases with slow progression, the hearing loss may remain unnoticed for a longer period of time. Therefore, prospective audiological assessment of yet asymptomatic mutation carriers will be required in the future to obtain more precise data. The audiogram shape and degree of hearing loss in *EYA4* deafness are variable. Most often it is described as mid-frequency hearing loss (“cookie bite” audiogram) of a mild to moderate degree at the time of onset. Subsequent hearing deterioration also affects the high frequencies resulting in the flat or down-sloping audiometric curve of a moderate to profound degree (Table 4). In our investigated family, the shape of audiogram was relatively constant (gently downsloping) since its onset. The severity of hearing loss based on pure tone average (0.5–4 kHz) in the five subjects with deafness duration of ≥20 years ranged from 43.75 dB to 77.5 dB, not yet fulfilling the traditional criteria for cochlear implantation.

The effect of *EYA4* truncating mutations on cardiac functions is often discussed due to the cardiomyopathy found in patients with large deletions comprising the variable domain [7, 30, 32]. However, despite the process of NMD is known now for almost four decades and was accepted to be the consequence of mutations introducing PTC [39], only Pfister et al. [40] suggested that the frameshift mutations in *EYA4* will cause NMD. In such a case it is irrelevant to discuss the localization of the so-called truncating mutations in different EYA4 domains. We agree with this view, as all the patients with PTC share the same phenotype without cardiac symptoms similarly to the individuals with a missense mutation, which is also the case of patients studied in this report. All of them had normal morphology and function of the heart, without any signs of dilation cardiomyopathy. This would argue for the haploinsufficiency as the mechanism of this dominantly inherited hearing loss. Thus, it is more probable, that the reported cardiomyopathy was caused by other genetic factors present in the large deleted regions or created by the event of deletion. The definitive answer could be attained by estimating the level of the *EYA4* mRNA transcripts in patient’s cells, but *EYA4* is not expressed in blood leukocytes making this test inaccessible without a biopsy.

In conclusion, a novel *EYA4* mutation, c.804G > C (p.Gln268His), was identified in a Slovak nonsyndromic ADNSHL family using WES and Sanger sequencing. This variant represents a nucleotide change at the last base pair of exon 10. We demonstrate in vitro that c.804G > C leads to exon 10 skipping resulting in frame-shift that introduces premature termination codon. To the best of our knowledge, this is the first *EYA4* mutation associated with ADSNHL in the Slavic Caucasian population. Regarding the auditory phenotype, most of the variability concerned the onset of hearing loss, whereas the shape of the audiometric curve and progression after clinical manifestation were relatively uniform.

## Abbreviations

ABR: Auditory brainstem responses
ADSNHL: Autosomal dominant sensorineural hearing loss
ARTA: Age-related typical audiogram
CT: Computer tomography
DFNA: Deafness nonsyndromic autosomal dominant
DFNB: Deafness nonsyndromic autosomal recessive
ECG: Electrocardiogram
*EYA4*: Eyes Absent Homolog 4
eya-HR: eya homologous region
eya-VR: eya variable region
MAF: Minor allele frequency
MRI: Magnetic resonance imaging
NMD: Nonsense-mediated mRNA decay
NSNHL: Nonsyndromic sensorineural hearing loss
PST: Proline-serine-threonine
PTC: Premature termination codon
SNHL: Sensorineural hearing loss
SNP: Single nucleotide polymorphism
VEMPs: Vestibular evoked myogenic potentials
WES: Whole exome sequencing
